# GmDBA1 and GmDBA2 Are Candidate NLR Genes Associated with Dwarfism and Enhanced Resistance to *Phytophthora sojae* in Soybean

**DOI:** 10.3390/plants15213296

**Published:** 2026-10-25

**Authors:** Ke Zhang, Jinlong Xu, Weihang Chen, Jingting Yang, Shizhen Zhao, Qing Lu, Fengning Xiang

**Affiliations:** 1 The Key Laboratory of Plant Development and Environmental Adaptation Biology, Ministry of Education, School of Life Sciences, Shandong University, Qingdao 266237, China; zkzk93@163.com (K.Z.); 18821641131@163.com (J.X.); cwh675892894@163.com (W.C.); yangjt17@163.com (J.Y.); 17862973989@163.com (S.Z.); 2 Shandong Key Laboratory of Precision Molecular Crop Design and Breeding, School of Life Sciences, Shandong University, Qingdao 266237, China; 3 Shandong Provincial Key Laboratory of Plant Stress Biology and Genetic Improvement, School of Life Sciences, Shandong University, Qingdao 266237, China

**Keywords:** soybean, *gmdba1*, NLR candidate genes, *Phytophthora sojae* P6497, transcriptomics, jasmonic acid, salicylic acid

## Abstract

*Phytophthora* root and stem rot (PRR), caused by *Phytophthora sojae* (*P. sojae*), severely reduces soybean yield. We isolated the recessive *gmdba1* mutant from an ethyl methanesulfonate (EMS)-mutagenized population of the soybean cultivar Hedou 12 (H12). The mutant exhibited dwarfism, reduced node number, increased branching, and crinkled leaves. Genetic mapping delimited the phenotype-associated locus to an approximately 154 kb interval on chromosome 18 containing 22 annotated genes. Resequencing and targeted sequence analysis prioritized two NLR candidates, *Glyma.18g226500* (*GmDBA1*, ***D****warfism* and ***B**asal **A**ctivation 1*) and *Glyma.18g226800* (*GmDBA2*), with four reported mutant-specific SNPs (three non-synonymous substitutions). Both *gmdba1* and one homozygous T_2_ carrying double-edited line in the two genes exhibited reduced disease symptoms following inoculation with *P. sojae* isolate P6497. These results support an association between perturbation of the *GmDBA1*/*GmDBA2*-containing locus, altered development, and enhanced resistance under the tested conditions. Transcriptomic and hormone analyses revealed constitutive activation of defense-related genes, jasmonic acid (JA) and salicylic acid (SA) contents in *gmdba1*, which likely explains the observed growth–defense tradeoff. Together, our findings characterize GmDBA1 and GmDBA2 suppresses basal resistance against *P. sojae*, providing a valuable genetic resource for breeding disease-resistant soybean varieties.

## 1. Introduction

Soybean (*Glycine max*) is an economically vital crop whose yield is severely constrained by diseases caused by oomycetes, fungi, bacteria, viruses and nematodes. Among these pathogens, *P. sojae* represents a particularly destructive soil-borne oomycete [1,2]. *P. sojae* can infect soybean throughout development, from seedling to mature stages. Its soil-borne nature and capacity to rapidly overcome host resistance pose major challenges for disease management [1,3]. Host genetic resistance remains the central strategy for PRR management. However, most classical Resistance to *P. sojae* (Rps) genes confer race-specific resistance, which can be compromised upon virulence shifts in pathogen populations [2,3,4]. Soybean resistance against *P. sojae* is thus increasingly regarded as a composite of major-effect Rps loci and quantitative resistance components, which creates opportunities to uncover resistance mechanisms with reduced dependence on specific pathogen genotypes [2,4,5,6]. Although abundant QTLs conferring quantitative resistance have been mapped in soybean, only a limited set of resistance genes has undergone cloning and functional characterization [7,8,9].

Nucleotide-binding leucine-rich repeat receptors (NLRs) are intracellular immune receptors that activate defense responses. NLRs can function as single receptors, genetically linked pairs, or components of receptor networks [10,11,12]. Experimentally characterized pairs involve functional interdependence and, in some cases, physical interaction [11,13]. Genomic proximity and sequence similarity alone are insufficient to establish such relationships. Misregulation of NLR signaling can lead to constitutive defense-associated phenotypes and growth penalties [14,15]. Other regulatory layers also contribute to immunity: cotton lncRNA7 and lncRNA2 regulate cell wall-associated defense genes and alter resistance to Verticillium wilt [16]. This provides a comparative example of immune regulation without implying that the same mechanism operates at the soybean *GmDBA1/GmDBA2* locus.

Plant immunity is closely connected to development, and sustained defense signaling can be associated with reduced vegetative growth and altered architecture [14,17]. Jasmonates regulate defense and developmental processes, whereas salicylic acid (SA) is a major component of immune signaling [18,19,20]. Their interactions depend on tissue, developmental stage, and pathogen context [21,22,23]. Autoactive NLR alleles can be accompanied by defense activation and growth defects [24], but concurrent hormone accumulation and dwarfism do not establish a causal relationship. Soybean studies have characterized JA- and SA-responsive transcriptional signatures and defense-associated regulators [25,26,27]. Combining such transcriptional information with direct hormone measurements can therefore help describe the physiological state of a mutant, while genetic or pharmacological intervention is needed to test whether hormones cause its developmental or resistance phenotypes.

In this study, we characterized the EMS-induced soybean mutant *gmdba1*, which exhibits dwarfism, altered leaf morphology, and reduced disease symptoms after inoculation with *P. sojae* P6497. Genetic mapping and sequence analysis prioritized two NLR candidates, *GmDBA1* and *GmDBA2*, within a chromosome 18 interval. A simultaneously edited line in the Williams 82 background showed similar developmental and disease phenotypes. Transcriptomic and hormone analyses were used to characterize associated changes in the mutant. We interpret these findings as evidence linking this candidate locus to growth and defense, while explicitly distinguishing this association from proof of NLR pairing, individual gene causality, or hormone-dependent mechanisms.

## 2. Results

### 2.1. Phenotypic Characterization of Gmdba1

**Figure 1:**
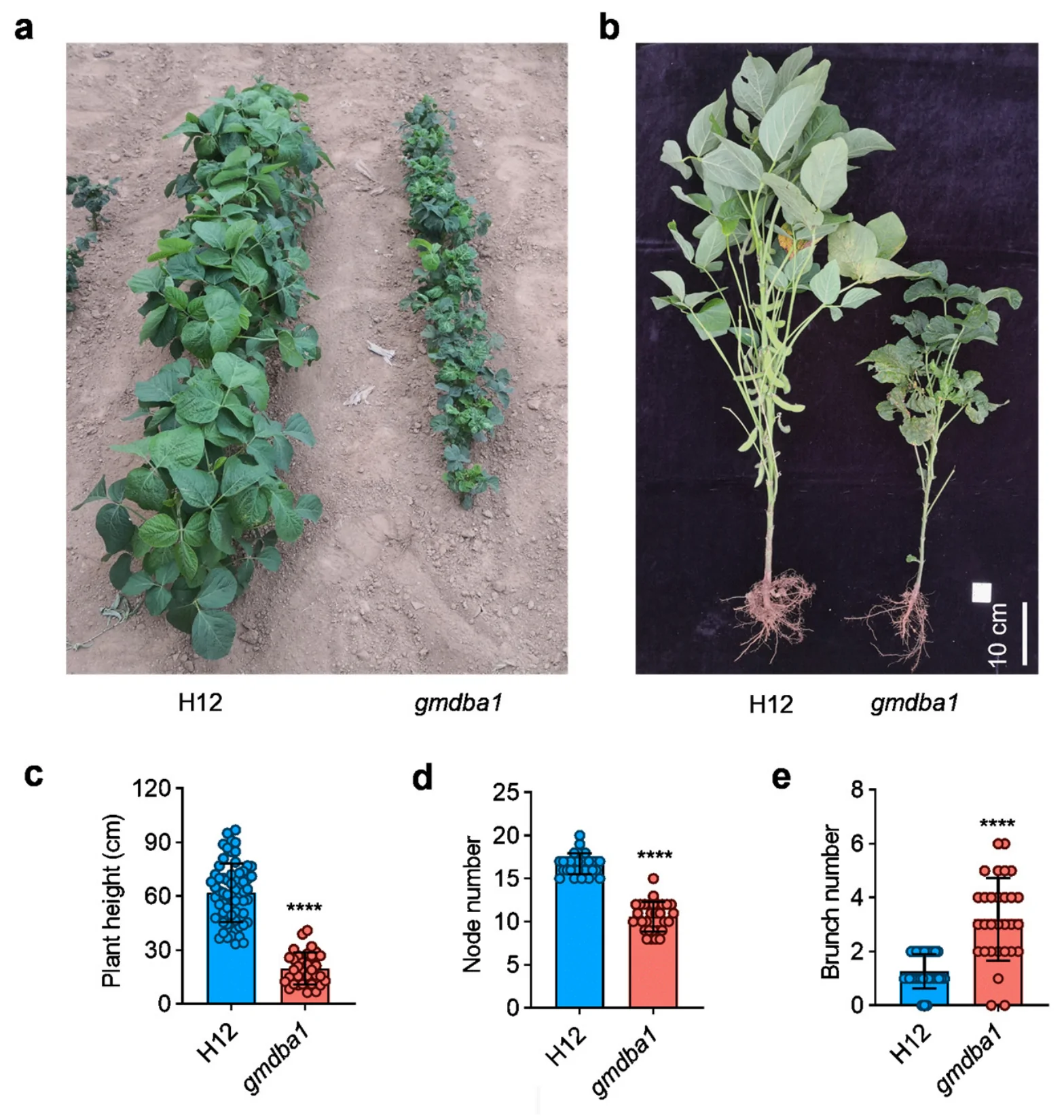
Phenotypic characterization of the *gmdba1* mutant under field and greenhouse conditions. (**a**) Representative field-grown plant photographs of wild-type H12 and *gmdba1* mutant. (**b**) Morphological comparison of whole-plant architecture between H12 and *gmdba1* under greenhouse conditions. Scale bar = 10 cm. (**c**–**e**) Quantitative measurement of agronomic traits, including plant height (**c**), node number (**d**), and branch number (**e**). Individual data points represent biological replicates. Data are presented as box-and-scatter plots. Statistical significance was determined by Student’s *t*-test; ****, *p* < 0.0001.

Through a forward genetic screen of ethyl-methanesulfonate (EMS)-mutagenized seeds derived from the high-yielding and stress-tolerant Chinese soybean cultivar H12, we identified the *gmdba1* mutant in the M_4_ populations based on its reduced plant height in the field compared with H12 plants. To assess the developmental impact of the mutation, we continuously monitored the growth dynamics of the *gmdba1* mutant alongside the H12 wild type (WT) under identical field conditions. Compared to H12, *gmdba1* exhibited a pleiotropic dwarf phenotype throughout both vegetative and reproductive stages (Figure 1a,b). Concurrently, during the V_4_ to V_10_ stages, the newly emerged leaves of the mutant displayed highly crinkled morphology (Figure 1a,b). Quantitative measurements revealed a >60% reduction in plant height (Figure 1c), and the mutant also showed reduced node number and increased branch number (Figure 1d,e).

### 2.2. Genetic Analysis and Fine Mapping of the Target Locus

**Figure 2:**
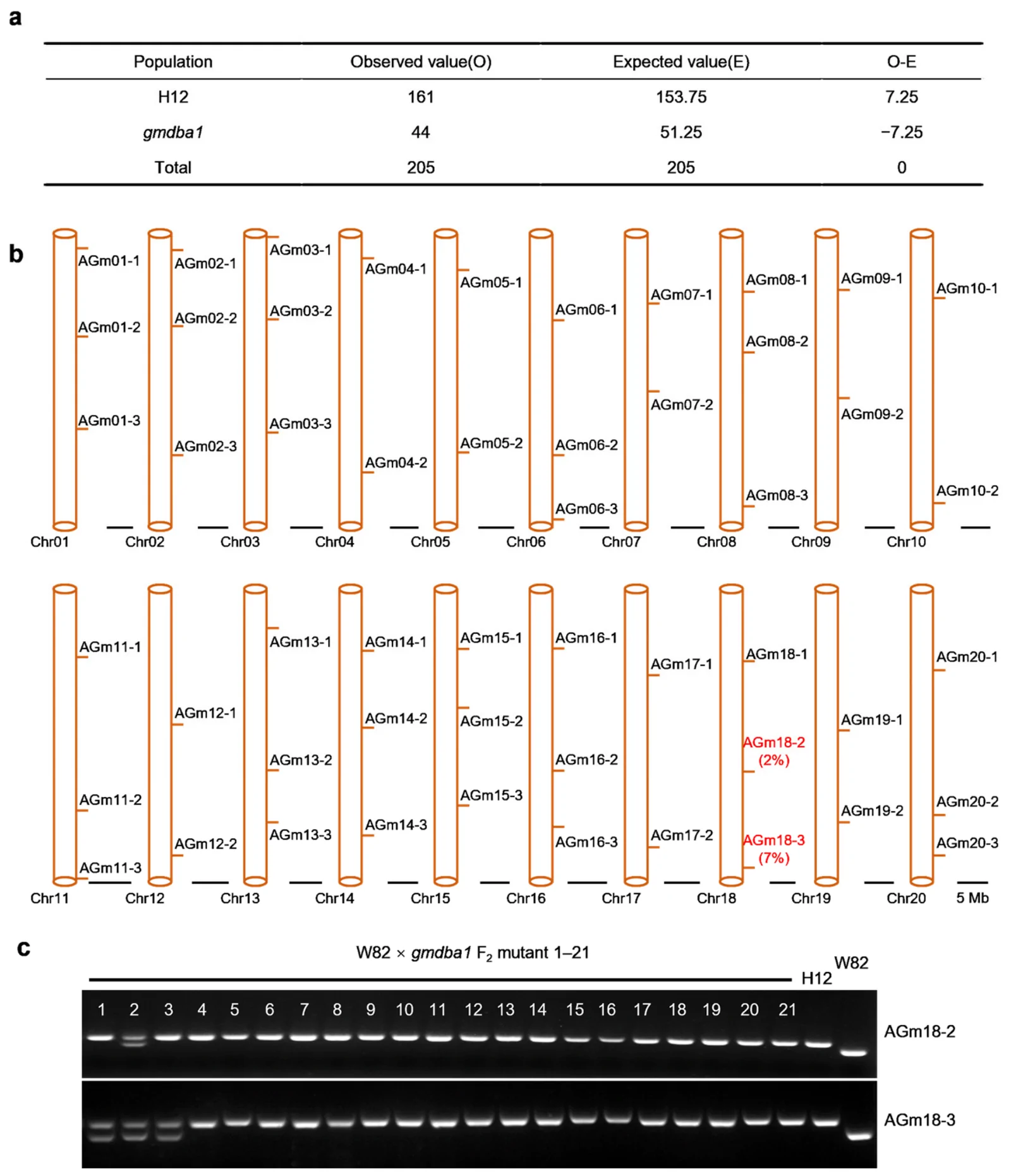
Genetic segregation and initial mapping of the *gmdba1*-associated locus. (**a**) Observed F_2_ counts were 161 wild-type-phenotype plants and 44 dwarf plants; exact expected counts under a 3:1 model are 153.75 and 51.25. Pearson χ^2^ = 1.367, df = 1, *p* = 0.242. (**b**) Genome-wide distribution of InDel markers across 20 soybean chromosomes. The reported recombination frequencies of AGm18-2 and AGm18-3 (Markers highlighted in red) on chromosome 18 are 2% and 7%, respectively. (**c**) Genotyping with AGm18-2 and AGm18-3. The F_2_ mutant samples are followed by the H12 and W82 parental controls, as labeled in the image. H12-type, W82-type, and heterozygous banding patterns represent a, b, and h marker genotypes, respectively.

To investigate inheritance, we analyzed an F_2_ population derived from a cross between Williams 82 (W82) and *gmdba1*. All F_1_ plants displayed a normal wild-type architecture, consistent with recessive inheritance. The F_2_ population contained 161 plants with a wild-type phenotype and 44 dwarf plants. A Pearson chi-square goodness-of-fit test without continuity correction gave χ^2^ = 1.367, df = 1, and *p* = 0.242. These counts are consistent with segregation at a single recessive locus (Figure 2a), which may contain more than one tightly linked gene.

**Figure 3:**
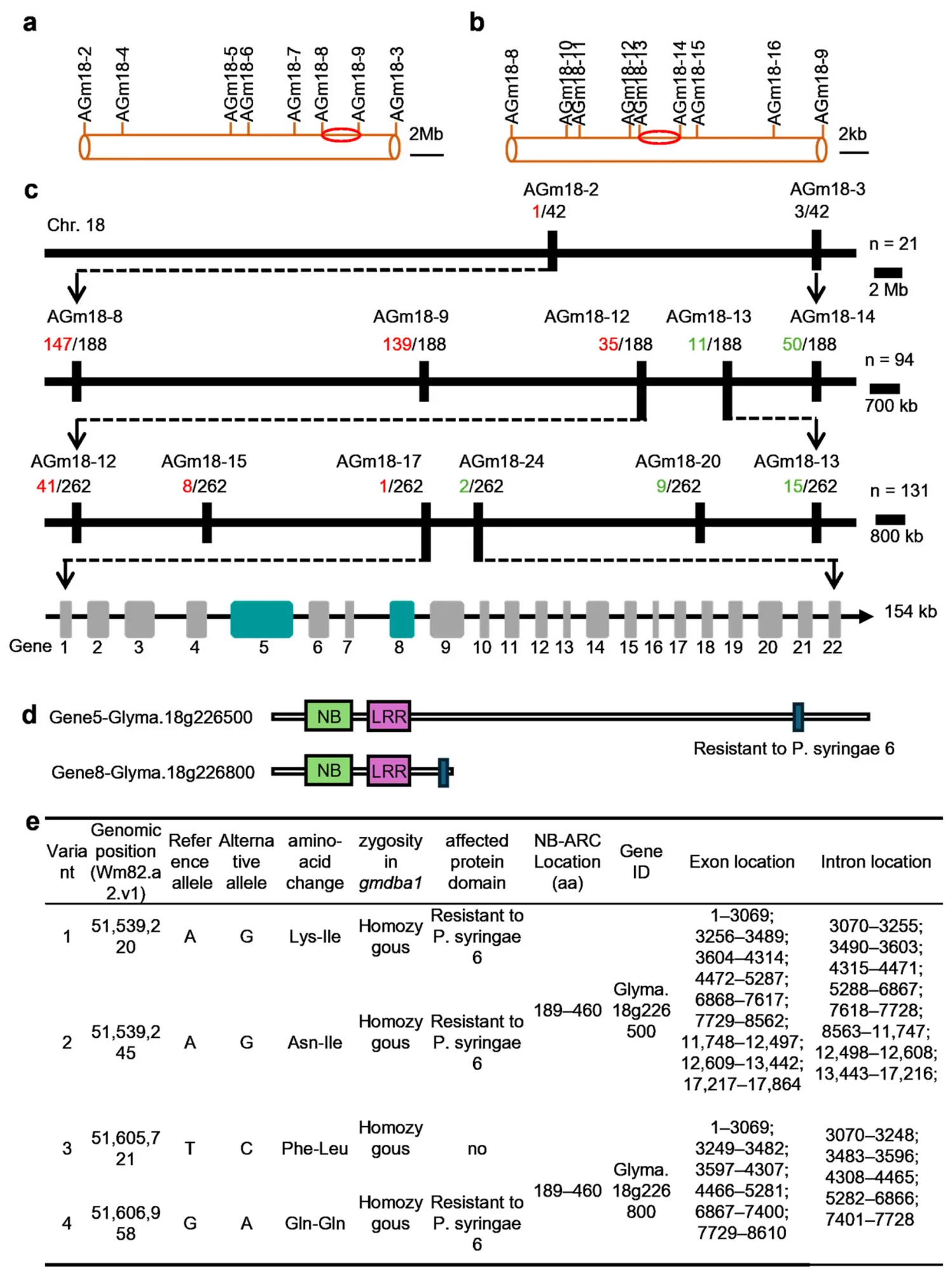
Fine-mapping of the *gmdba1* locus and prediction of candidate genes. (**a**,**b**) Schematic diagram of preliminary genetic interval delimitation on soybean chromosome 18 using molecular markers. Red ellipses denote the primary candidate genomic region. (**c**) High-resolution fine-mapping of the *gmdba1* locus using recombinant individuals from F_2_ and F_3_ segregating populations. Numbers above each marker represent the number of recombinant events over total screened progeny. The horizontal black bars represent the progressively narrowed genetic intervals. The final mapping interval spans a 154 kb physical region containing 22 annotated open reading frames. The scale bars indicate corresponding physical distances. The red/green numbers indicate recombination frequencies between markers and the target locus. Arrows represent the mapping interval narrowed down by recombinant screening. (**d**) Domain architecture of two candidate NB-LRR resistance-related proteins within the fine-mapped interval. NB, nucleotide-binding domain; LRR, leucine-rich-repeat domain. Gene identifiers (*Glyma.18g226500* and *Glyma.18g226800*) are annotated for each protein. The colored boxes in gene structures denote conserved domains. (**e**) Genomic variation information of four SNP sites identified in the *gmdba1* mutant. Reference allele, alternative allele, corresponding gene, exon-intron positions and the amino-acid range of NB-ARC domain are listed.

**Figure A1:**
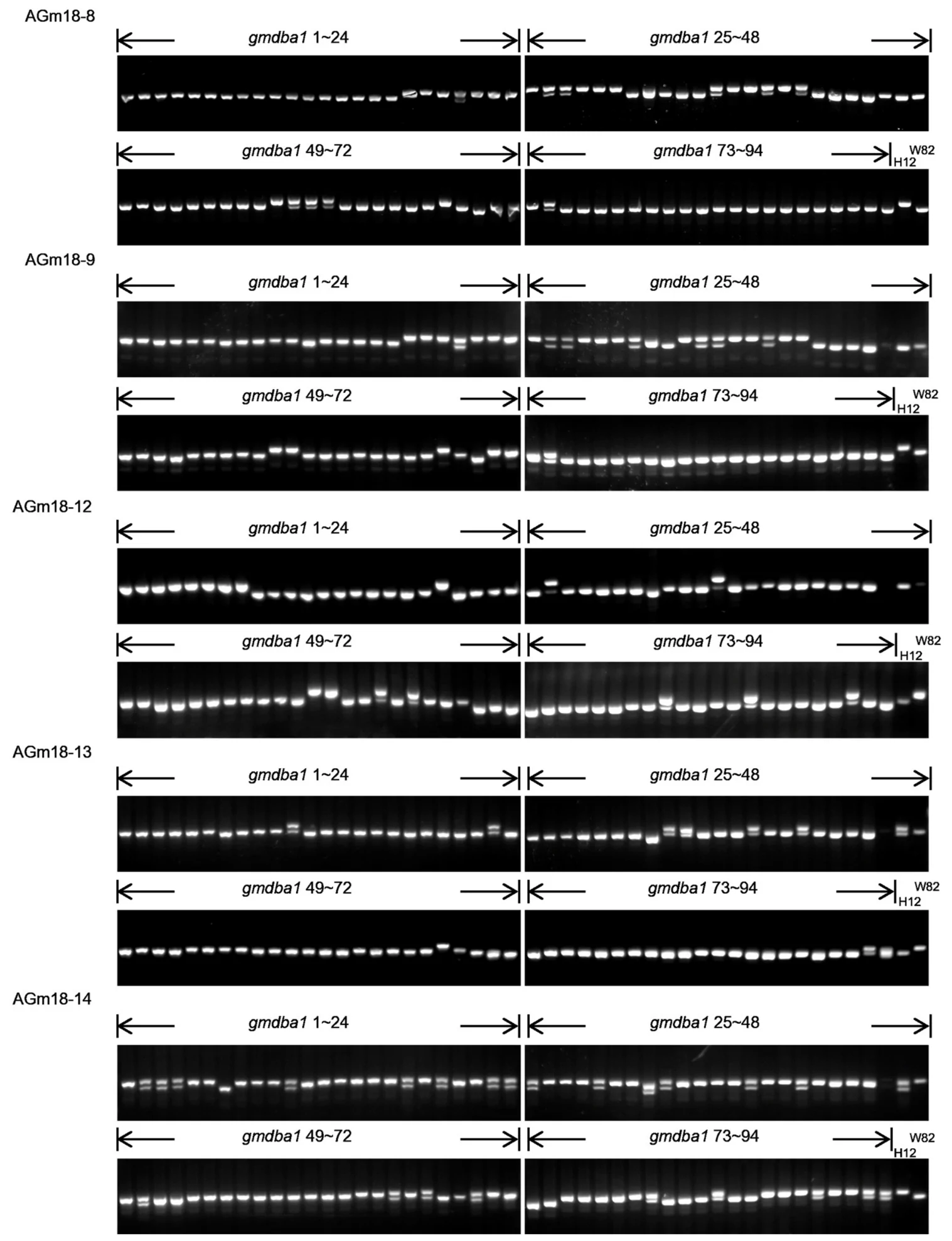
Genotyping PCR-gel analysis of 94 F_2_ mutant individuals used for fine-mapping. Agarose gel electrophoresis showing genotyping results using five polymorphic molecular markers (AGm18-8, AGm18-9, AGm18-12, AGm18-13 and AGm18-14) across 94 *gmdba1* mutant plants. Individual mutant samples were divided into four groups (1–24, 25–48, 49–72, 73–94). The arrows denote the boundaries of each sample group. Wild-type controls H12 and W82 were included for allelic comparison.

**Figure A2:**
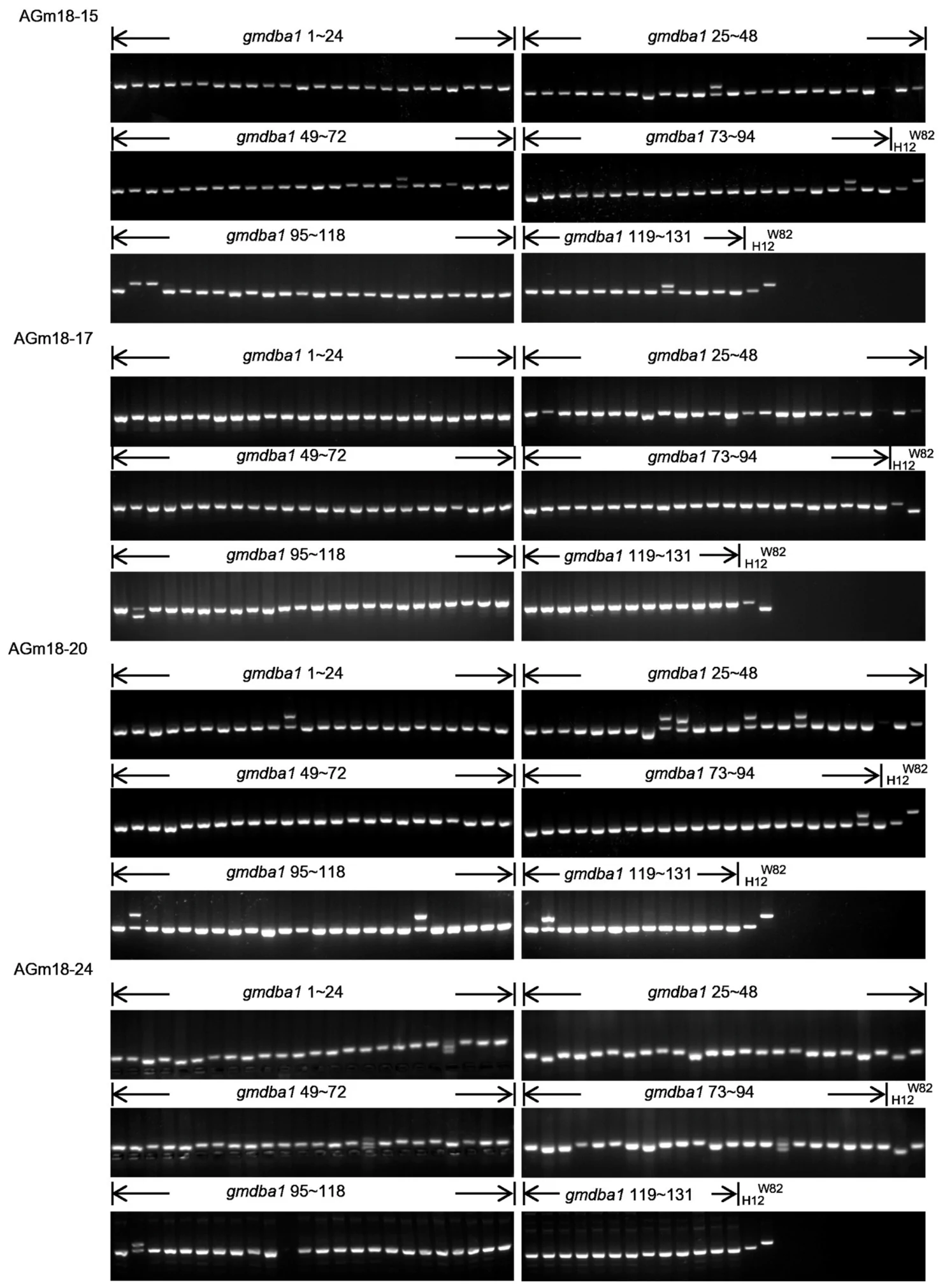
Genotyping PCR-gel assays of 131 F_2_ mutant individuals for high-resolution fine-mapping. Agarose gel electrophoresis displaying genotyping profiles obtained with four polymorphic markers (AGm18-15, AGm18-17, AGm18-20 and AGm18-24). A total of 131 *gmdba1* mutant plants were divided into six sample groups (1–24, 25–48, 49–72, 73–94, 95–118, 119–131). The arrows denote the boundaries of each sample group. Wild-type controls H12 and W82 were loaded as reference allelic controls.

**Figure A3:**
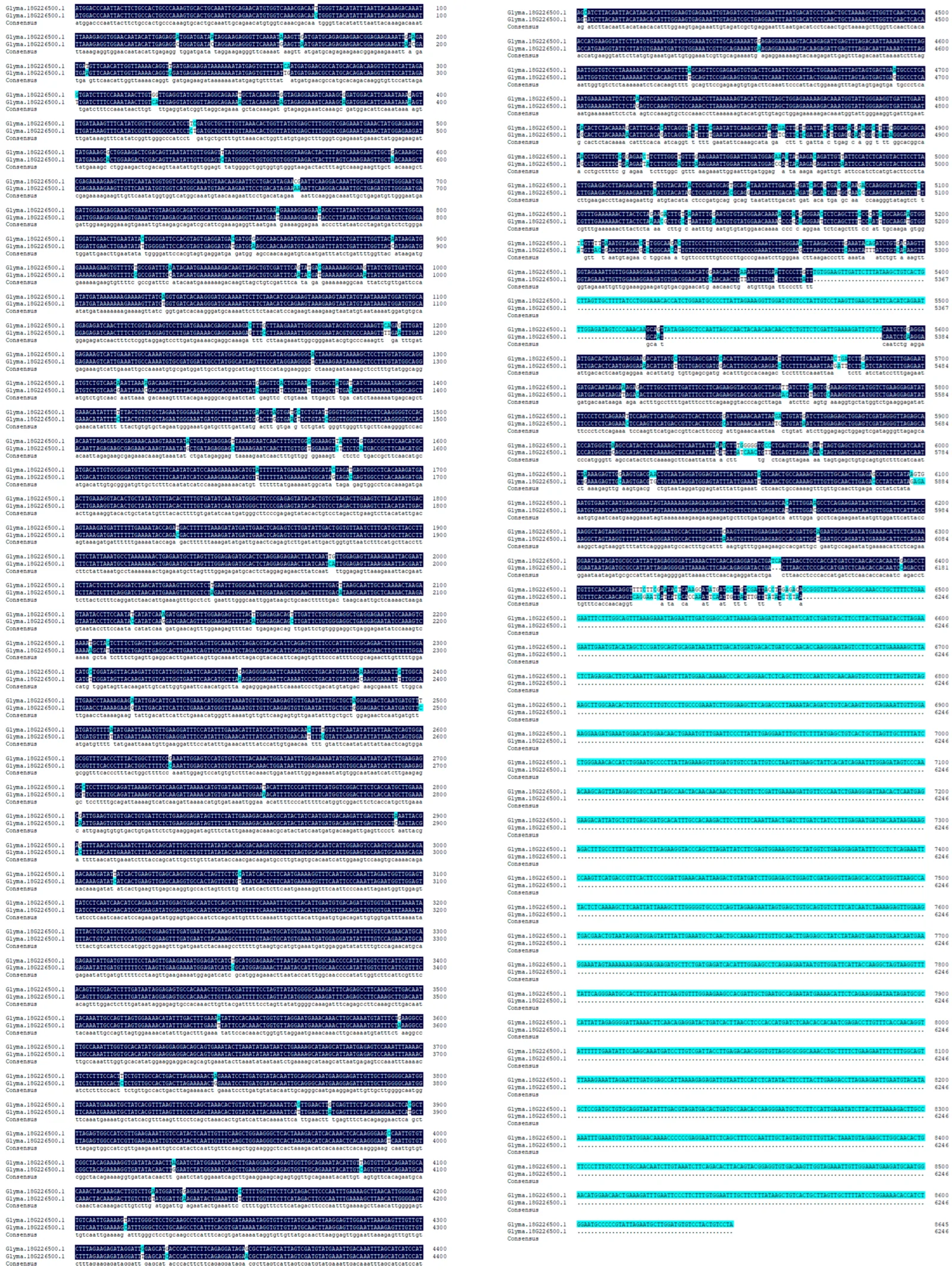
CDS sequence comparison between *GmDBA1* (*Glyma.18g226500.1*) and *GmDBA2* (*Glyma.18g226800.1*). Dark blue indicates identical nucleotide sites; light blue indicates divergent nucleotide sites; dotted lines represent sequence gaps.

**Figure A4:**
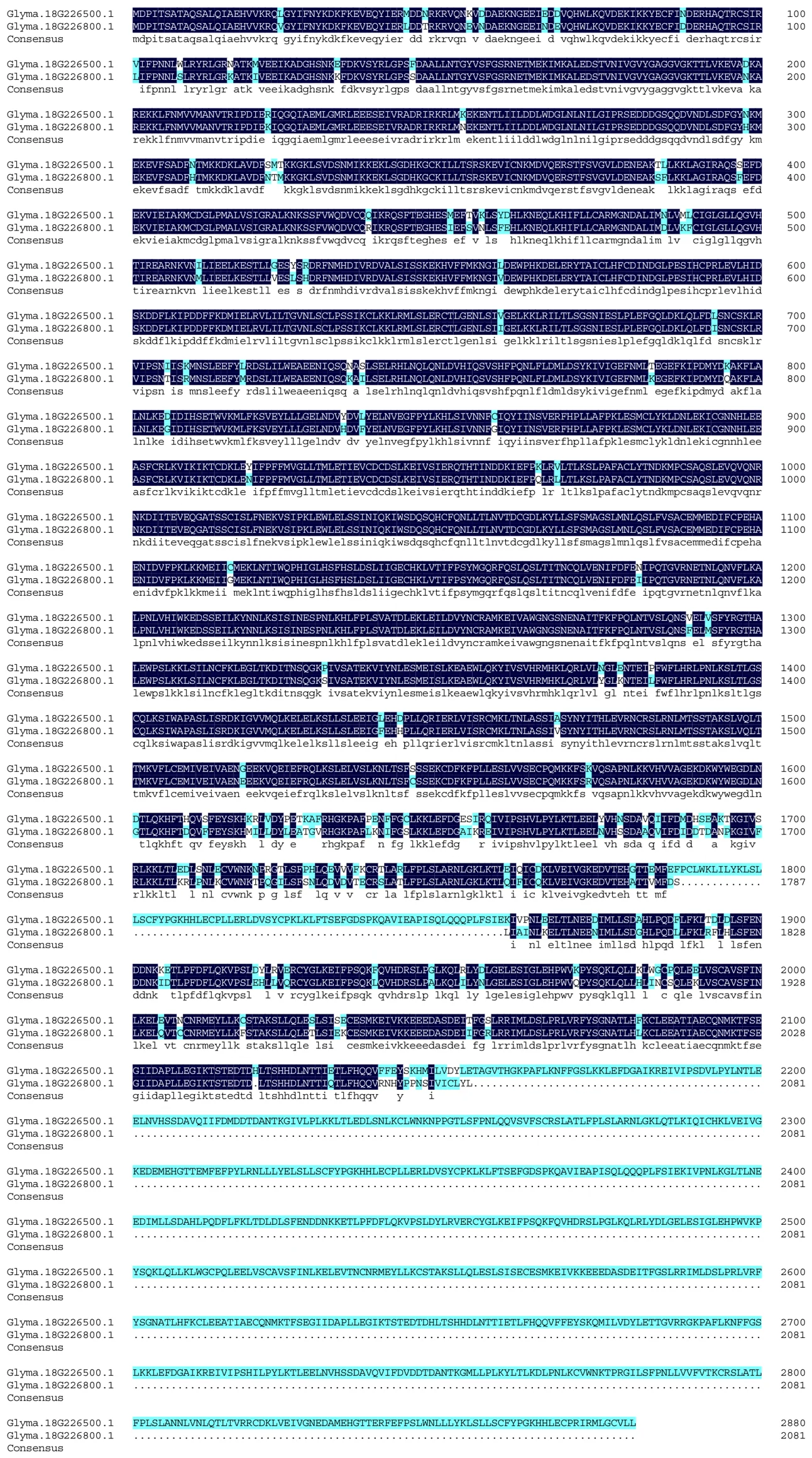
Predicted protein sequence comparison between GmDBA1 (*Glyma.18g226500.1*) and GmDBA2 (*Glyma.18g226800.1*). Dark blue indicates identical amino acid residues; light blue indicates divergent amino acid residues; dotted lines represent alignment gaps.

Initial genome-wide marker screening implicated chromosome 18; the low marker recombination frequencies reported for AGm18-2 and AGm18-3 (2% and 7%, respectively; Figure 2b) directed subsequent fine-mapping to this chromosome. Screening 94 mutant F_2_ plants delimited an approximately 2 Mb interval between AGm18-8 and AGm18-14 (Figure 3a,b and Figure A1). Further analysis of 131 mutant F_2_ plants narrowed the interval to approximately 154 kb between AGm18-17 and AGm18-24 (Figure 3c and Figure A2). The interval contains 22 annotated genes (Table S2). To further validate these candidates, we performed individual gene cloning and Sanger sequencing for all 22 annotated genes residing in this genomic region. Sequence alignment showed that the coding sequences of 20 out of the 22 genes were identical between the *gmdba1* mutant and wild-type H12, and these genes were therefore excluded as causal candidates (Table S2). Pooled whole-genome resequencing revealed that four mutant-specific SNPs (Table S5) were consistently detected in the remaining two genes (Gene 5: GmDBA1, and Gene 8: GmDBA2). Among these four SNPs, three were non-synonymous substitutions leading to amino acid changes, while one was a synonymous mutation without altering the encoded amino acid (Figure 3e). Both two proteins contain predicted NB-ARC and LRR domains (Figure 3d,e). Sequence characterization showed that GmDBA1 had a full-length sequence of 43,083 bp with nine exons and eight introns, encoding a protein of 2882 amino acids, whereas GmDBA2 was 8610 bp in length with six exons and five introns, encoding a 2082-amino-acid protein (Figure 3e). Despite these structural differences, the two genes exhibited extremely high sequence similarity, with 69% identity in their coding sequences (Figure A3) and 87% identity at the amino acid level (Figure A4). Taken together, these two genes were prioritized as the most likely candidate genes responsible for the dwarf and wrinkled-leaf phenotype of the *gmdba1* mutant.

**Figure 4:**
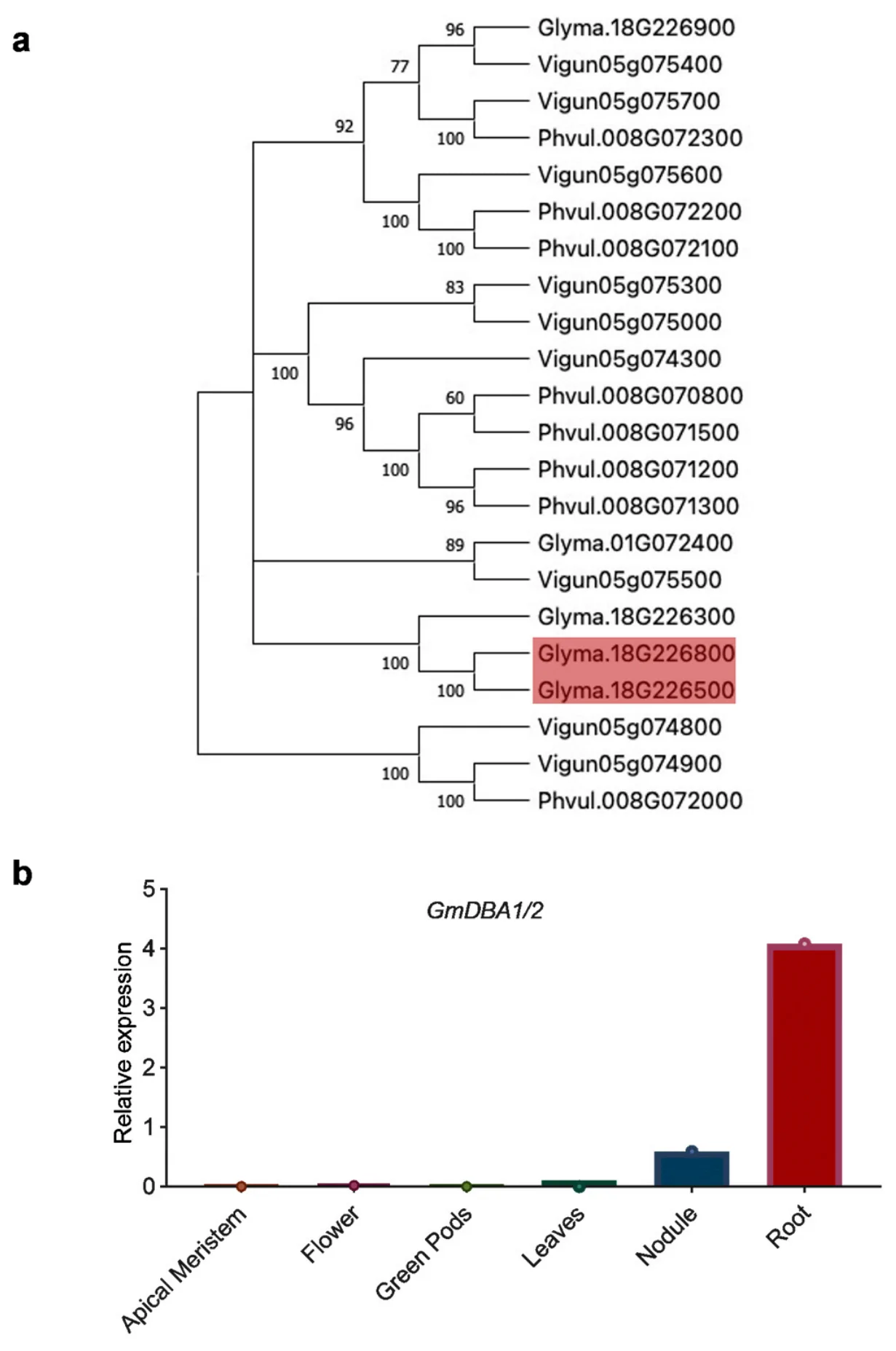
Phylogenetic relationships and tissue-associated expression of GmDBA1/GmDBA2. (**a**) Tree of homologous proteins from *Glycine max*, *Phaseolus vulgaris*, and *Vigna unguiculata*. Numerical node labels are shown as in the original analysis; the GmDBA1/GmDBA2 (Sequences highlighted in red correspond) node has a displayed support value of 1000. (**b**) Relative RT-qPCR expression in apical meristem, flower, green pods, leaves, nodule and root.

To further dissect the evolutionary relationships of these two paralogous genes within legumes, a phylogenetic tree was constructed using homologous protein sequences from soybean, common bean, and cowpea. Phylogenetic reconstruction classified legume homologous proteins into several independent sub-clades. GmDBA1 (*Glyma.18g226500*) and GmDBA2 (*Glyma.18g226800*) clustered closely within a single monophyletic branch, suggesting that they are paralogous genes derived from a recent common ancestor in soybean (Figure 4a).

### 2.3. Tissue-Specific Expression

To explore the potential biological functions of *GmDBA1/2*, we characterized their transcript accumulation patterns across multiple soybean tissues using qRT-PCR. The results demonstrated that both genes maintained relatively low expression in the apical meristem, flower, green pods, leaves, and nodules. By contrast, substantially higher transcript levels were detected in whole root tissues, exhibiting approximately 4-fold higher expression compared with other tested tissues (Figure 4b). Taken together, these data indicate that *GmDBA1/2* are preferentially expressed in soybean roots, aligning with the biological reality of *P. sojae*, a destructive soil-borne root pathogen that primarily infects root tissues.

### 2.4. Reduced Disease Symptoms in Gmdba1 Following P6497 Inoculation

**Figure 5:**
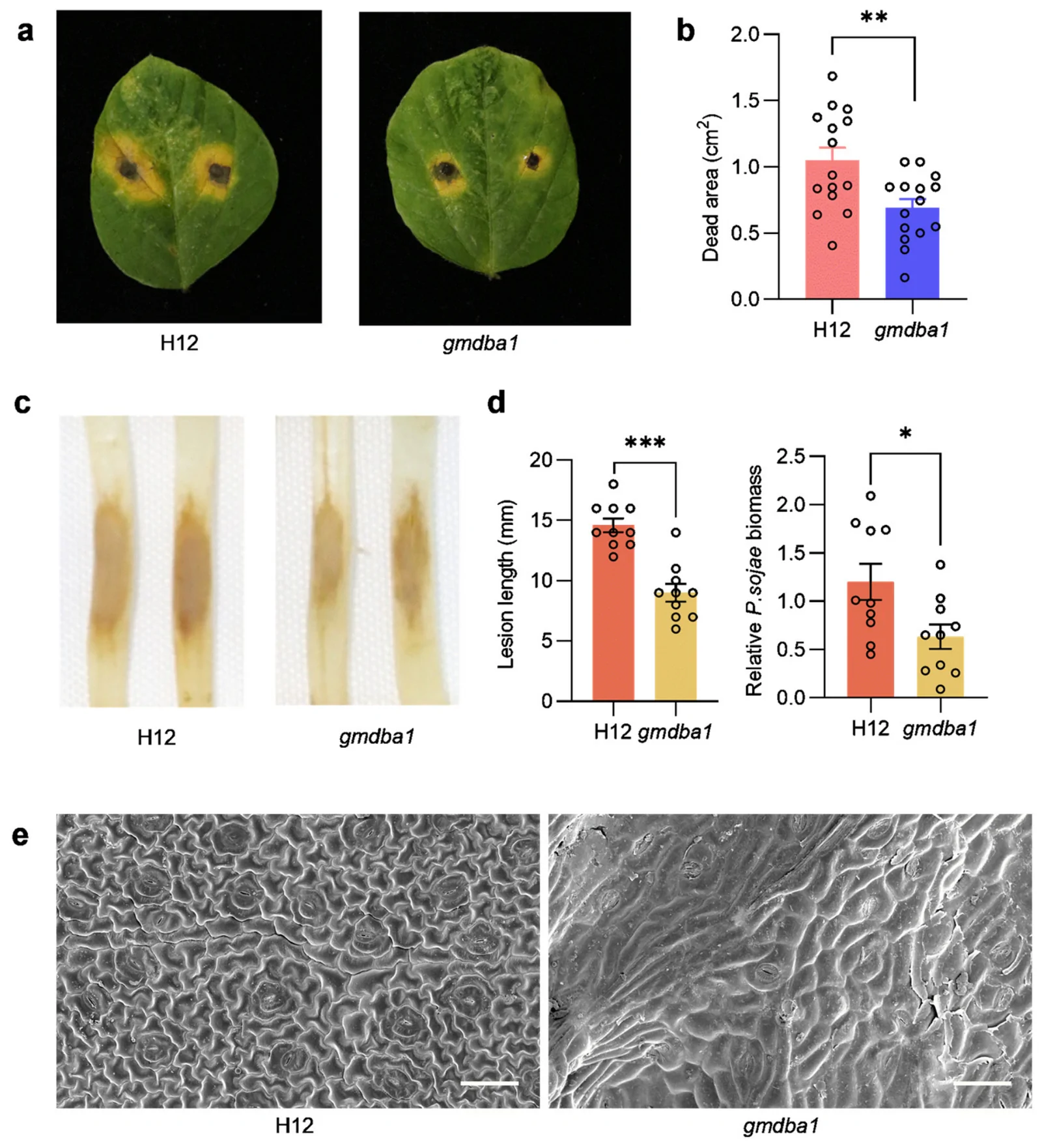
Disease-resistance phenotype and cellular morphological comparison between wild-type H12 and *gmdba1* mutant. (**a**) Representative leaf photographs showing lesion symptoms of H12 and *gmdba1* after *P. sojae* inoculation. (**b**) Quantification of leaf necrotic dead area. Each dot indicates an independent biological replicate. Data represent mean ± SD; **, *p* < 0.01 (Student’s *t*-test). (**c**) Hypocotyl lesion phenotypes of H12 and *gmdba1* after *P. sojae* inoculation. (**d**) Quantitative measurement of hypocotyl lesion length and relative *P. sojae* biomass. Each dot indicates an independent biological replicate. Data represent mean ± SD; ***, *p* < 0.001; *, *p* < 0.05 (Student’s *t*-test). (**e**) Scanning electron microscopy (SEM) images of leaf epidermal cells from H12 and *gmdba1*. Scale bars are indicated in each micrograph.

Given that *GmDBA1/2* encode NB-ARC-LRR-type immune-related proteins, we hypothesized that morphological alterations triggered by the *gmdba1* mutation are coupled with plant immune activation. To verify this hypothesis, detached leaves from seedlings were inoculated with a virulent *P. sojae* isolate. At 7 days post-inoculation (dpi), wild-type H12 leaves exhibited obvious disease symptoms with expanded water-soaked necrotic lesions. In contrast, *gmdba1* leaves exhibited a robust hypersensitive response, restricting pathogen spread within small localized necrotic regions around inoculation sites (Figure 5a). Quantitative measurement of lesion dead area further validated that the *gmdba1* mutation significantly enhances resistance against *P. sojae* (Figure 5b). Meanwhile, we also subjected the lower hypocotyls of soybeans to Phytophthora infection. The results were consistent with the previous ones. The lesion size on *gmdba1* hypocotyls was significantly smaller than that on H12 hypocotyls. (Figure 5c,d). To explore the cellular features linked to this phenotypic difference, scanning electron microscopy (SEM) was performed to observe adaxial leaf epidermal morphology. SEM observations revealed irregular cuticular wax deposition on the leaf surface of the *gmdba1* mutant, accompanied by altered epidermal cell morphology (Figure 5c).

### 2.5. Phenotypes of a Line Carrying Simultaneous CRISPR/Cas9 Edits

**Figure 6:**
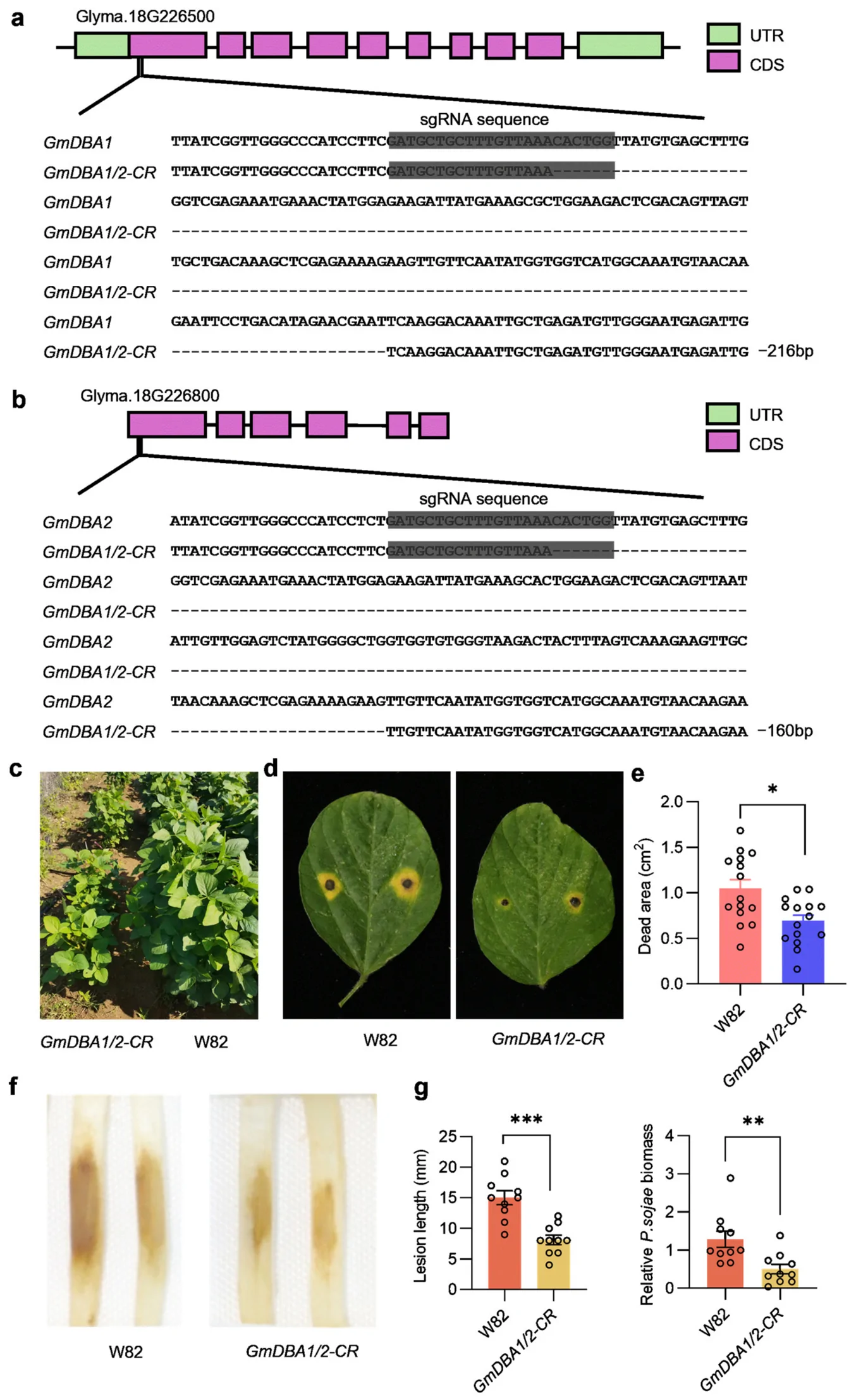
CRISPR-Cas9-mediated gene editing validation of *GmDBA1/2*. (**a**,**b**) Gene-structure diagrams and sequence alignment showing CRISPR-Cas9-induced fragment deletion in *Glyma.18g226500* (**a**) and *Glyma.18g226800* (**b**). Green boxes represent UTR regions, purple boxes indicate CDS exons. The sgRNA target site is highlighted in black background. Sequence alignment displays the nucleotide differences between wild-type *GmDBA1/2* and edited *GmDBA1/2-CR* line; dashed lines denote deleted sequences, and a 216 bp and 160 bp fragment deletion is indicated. (**c**) Whole-plant morphological phenotypes of CRISPR-edited *GmDBA1/2-CR* plants and wild-type W82 under field conditions. (**d**) Representative leaf disease-lesion photographs of W82 and *GmDBA1/2-CR* after *P. sojae* P6497 inoculation. (**e**) Quantification of leaf dead lesion area in W82 and *GmDBA1/2-CR*. Each dot corresponds to one biological replicate. Data are presented as mean ± SD; *, *p* < 0.05 (Student’s *t*-test). (**f**) Representative hypocotyl disease symptoms of wild-type W82 and *GmDBA1/2* double-edited line (*GmDBA1/2-CR*) after inoculation with *P. sojae* P6497. (**g**) Quantitative analysis of hypocotyl lesion length and relative *P. sojae* biomass in W82 and *GmDBA1/2-CR* plants. Each dot represents one independent biological replicate. Data are presented as mean ± SD; ***, *p* < 0.001; **, *p* < 0.01 (Student’s *t*-test).

To provide direct genetic evidence that perturbation of this paired NLR module causes the observed phenotypes, we utilized the CRISPR/Cas9 system to simultaneously edit *GmDBA1* and *GmDBA2* in the susceptible W82 background. A homozygous T_2_ double-knockout line was obtained and verified by Sanger sequencing (Figure 6a,b). Sequencing confirmed a 216 bp deletion in *GmDBA1*, resulting in an in-frame deletion of 72 amino acids; a 160 bp deletion in *GmDBA2* leads to premature termination of translation. Under standard growth conditions, these double-edited lines precisely phenocopied the *gmdba1* mutant, exhibiting severe dwarfism, shortened internodes, and crinkled leaves (Figure 6c). Furthermore, pathogen inoculation assays demonstrated that these knockout lines gained significant resistance to *P. sojae*, closely resembling the immune response of the original *gmdba1* mutant, while the untransformed W82 controls remained highly susceptible (Figure 6d–g). These results confirm that simultaneous disruption of these two NLR modules induces both developmental defects and enhanced immunity. However, this double-knockout experiment alone cannot resolve whether the phenotype arises primarily from loss of *GmDBA1* or loss of *GmDBA2*, requires loss of both genes, or involves compensatory interactions between the two NLR partners.

### 2.6. Defense-Associated Transcriptional Changes in Gmdba1

**Figure 7:**
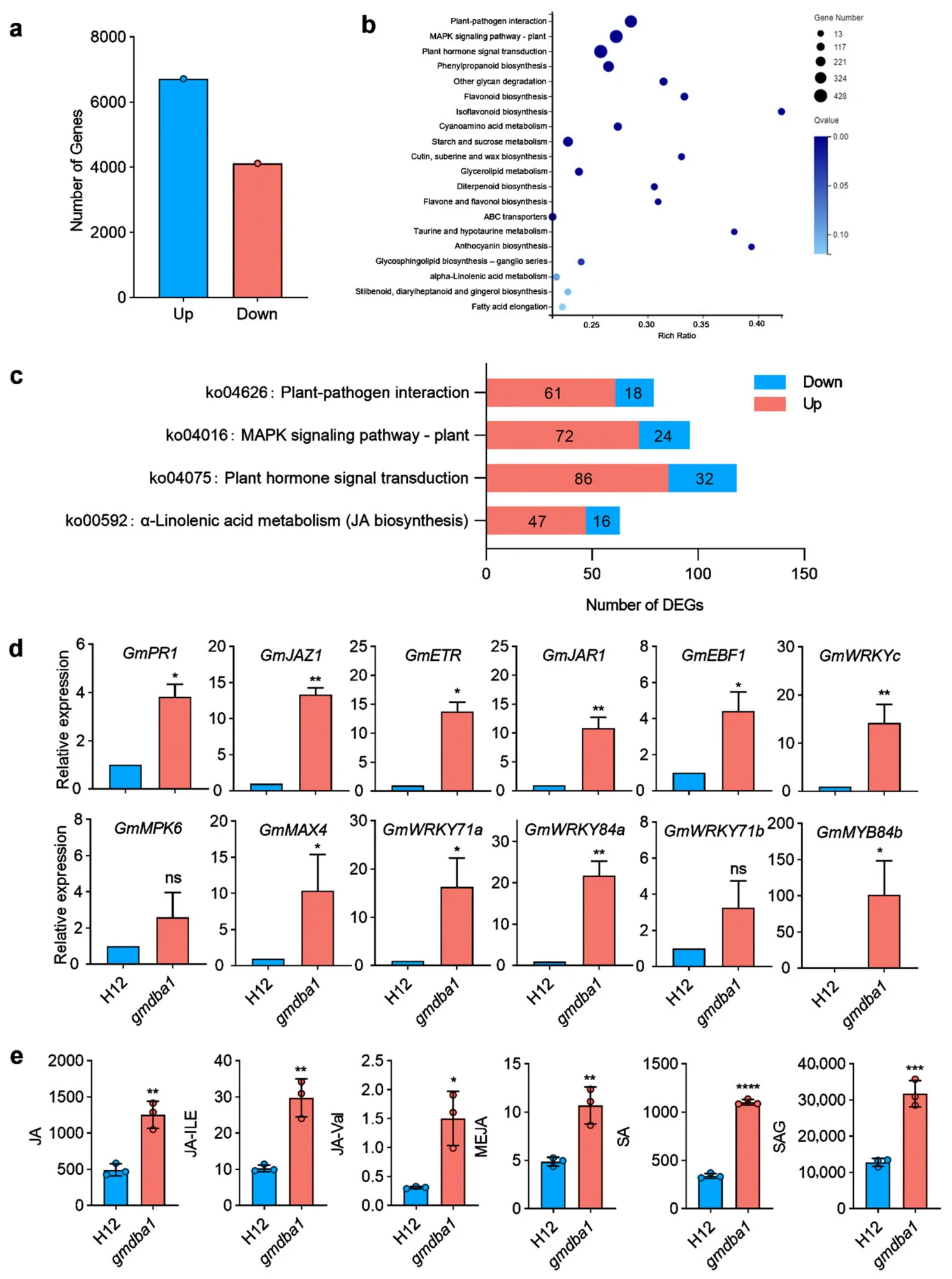
Transcriptomic differences and hormone abundance in the EMS-derived *gmdba1* mutant and H12. (**a**) Number of significantly up- and down-regulated differentially expressed genes (DEGs) between *gmdba1* and H12 control plants. (**b**) KEGG enrichment scatter plot of DEGs. Rich factor represents the ratio of DEG number to total gene number enriched in each pathway; dot size corresponds to gene count, and color indicates adjusted *p*-value. Defense-associated pathways including plant-pathogen interaction, MAPK signaling, and phytohormone signal transduction were highly enriched. (**c**) Four representative pathways were selected: α-linolenic acid metabolism (ko00592, JA biosynthesis), plant hormone signal transduction (ko04075), MAPK signaling pathway-plant (ko04016), and plant-pathogen interaction (ko04626). DEGs were filtered by thresholds of |log_2_FC| ≥ 1 and adjusted *p*-value ≤ 0.001. Red and blue segments indicate up-regulated and down-regulated genes, respectively. (**d**) qRT-PCR validation of representative DEGs associated with JA/SA defense signaling. H12 served as wild-type control; ns, no significant difference; *p* < 0.05, * *p* < 0.05, ** *p* < 0.01 (Student’s *t*-test). (**e**) Quantification of endogenous phytohormone contents, including JA, JA-Ile, JA-Val, MEJA, SA and SAG in H12 and *gmdba1* materials. Data are presented as mean ± SD; * *p* < 0.05, ** *p* < 0.01, *** *p* < 0.001, **** *p* < 0.0001 (Student’s *t*-test).

**Figure A5:**
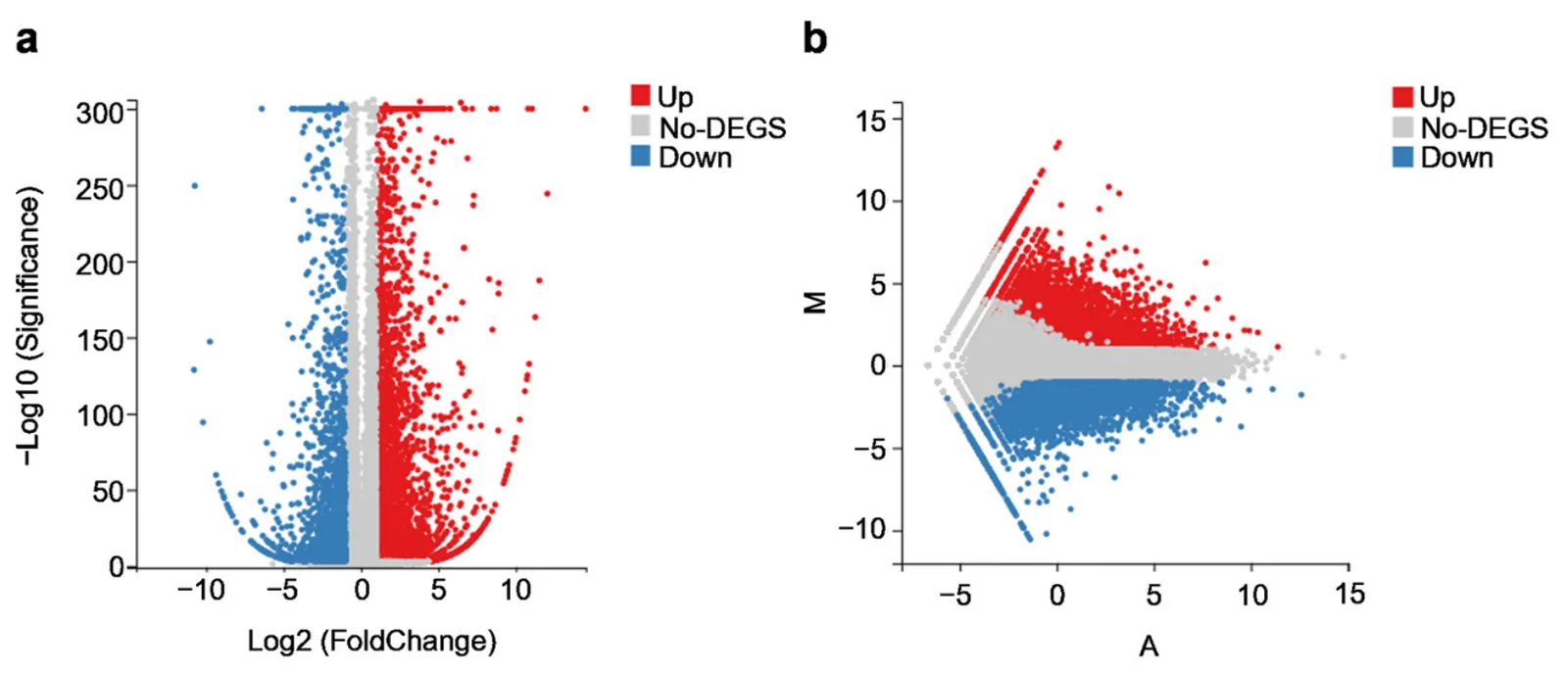
Overview of differentially expressed genes (DEGs) between *gmdba1* mutant and wild-type H12 based on transcriptome analysis. (**a**) Volcano plot illustrating global gene-expression changes. The x-axis represents log_2_ (fold-change), and the y-axis indicates −log_10_ (Significance). Red dots denote significantly up-regulated genes, blue dots represent significantly down-regulated genes, and gray dots indicate genes with no significant expression changes (no-DEGS). (**b**) MA plot showing the distribution of DEGs. The x-axis corresponds to the mean expression value (A), and the y-axis represents log_2_ (fold-change) (M). Red, blue, and gray dots represent up-regulated, down-regulated, and non-differentially expressed genes, respectively.

To uncover the molecular networks operating downstream of the mutated two NLR, we performed comparative RNA-seq analysis on V_6_ stage apical shoot tissues, while hormone quantification was conducted on fully expanded leaves at the identical developmental stage. The transcriptomic profiling identified differentially expressed genes (DEGs) between H12 and *gmdba1* (Figure 7a and Figure A5). KEGG pathway and Gene Ontology (GO) enrichment analyses showed that these DEGs were highly enriched in biological processes related to plant–pathogen interaction, MAPK signaling cascades, and JA and SA hormone biosynthesis and signal transduction (Figure 7b).

Notably, the transcriptomic data revealed constitutive activation of basal defense networks (Figure 7b). We therefore further examined several functionally important defense genes by qRT-PCR, including the canonical marker gene for SA-mediated plant defense responses *GmPR1* [28], the bioactive JA-Ile synthesize gene *GmJAR1* [29], and the well-known master regulators for immune transcriptional reprogramming WRKY and MYB transcription factors [30]. All of these genes were significantly up-regulated in *gmdba1* relative to H12 (Figure 7c,d). This observation suggests that the mutation drives a constitutive defense-on state independent of pathogen effectors.

### 2.7. Enhanced Accumulation of Endogenous JA- and SA-Derived Defense Phytohormones in Gmdba1

Given the prominent transcriptional activation of JA- and SA-signaling modules in *gmdba1*, we further quantified endogenous defense-related phytohormone accumulation. The contents of JA, its bioactive conjugates JA-Ile and JA-Val, and methyl jasmonate (MeJA) were significantly elevated in *gmdba1* relative to H12 (Figure 7e and Table S4). Consistently, SA and its storage form salicylic acid 2-O-β-D-glucoside (SAG) also exhibited markedly higher abundance in mutant tissues (Figure 7e and Table S4). These biochemical measurements demonstrated that disruption of the *GmDBA1/2* leads to the accumulation of both JA- and SA-class defense phytohormones (Figure 7d). Combined with transcriptomic data, these findings indicate that the *GmDBA1/2* participates in restraining SA- and JA-dependent immune outputs under basal conditions. Mutation of this module triggers hormone overaccumulation and activation of downstream immune signaling, accompanied by constitutive resistance and severe developmental defects.

## 3. Discussion

### 3.1. Evidence Implicating the GmDBA1/GmDBA2-Containing Locus in Development and P6497 Resistance

Genetic mapping delimited the causal locus responsible for the recessive mutant phenotype to a 154 kb interval on chromosome 18, which harbors two tandem nucleotide-binding leucine-rich repeat (NLR) genes, *GmDBA1* and *GmDBA2*. Although marker-assisted linkage analysis constrained this candidate interval, positional cloning alone is insufficient to resolve individual gene functions within multi-NLR clusters. Here, genomic sequence variations in the mutant, coupled with consistent phenotypic defects recapitulated in a CRISPR/Cas9-edited *gmdba1 gmdba2* double-knockout line in the Williams 82 background, provide convergent genetic evidence that this NLR locus regulates both plant development and resistance to *P. sojae*.

Nonetheless, several mechanistic questions warrant further investigation. Because our reverse-genetic validation utilized a simultaneous double-knockout, the current data cannot distinguish the discrete contributions of *GmDBA1* and *GmDBA2*, nor establish whether they operate as a canonical sensor–helper NLR pair or cooperatively in a convergent signaling module [11,13]. Generating single-gene knockouts, reciprocal genetic complementation lines, and testing physical or functional heterodimerization will be essential to decipher their specific molecular hierarchy. Additionally, phenotypic evaluations in this study were conducted on a single double-edited line; analyzing multiple independent edited alleles and stable complementation lines will fully rule out potential somaclonal variation or off-target effects.

Finally, pathogen assays demonstrated enhanced resistance of the mutant and edited plants specifically against *P. sojae* isolate P6497. Plant disease resistance is frequently race- or isolate-specific, and it remains to be determined whether this locus confers broad-spectrum resistance against a wider collection of *P. sojae* pathotypes or other agronomically important soybean pathogens. Systematically testing an expanded panel of virulent isolates alongside single-gene genetic models will define the exact resistance spectrum and mode of action of this immune locus.

### 3.2. Transcriptomic and Hormone Associations with the Growth-Defense Phenotype

Plant immunity and development are closely intertwined, and sustained defense activation often causes growth-defense trade-offs whereby auto-immunity enhances disease resistance while impairing plant growth [31]. Our RNA-seq data showed that numerous defense-related genes and key JA- and SA-signaling components were significantly up-regulated in the *gmdba1* mutant, suggesting sustained self-activation of these pathways that accounts for its enhanced resistance.

JA and SA are dual-function phytohormones governing both immunity and plant morphogenesis [18,20]. These two hormones can exert either antagonistic or synergistic regulatory effects on plant disease resistance, depending on plant species, pathogen lifestyle, and the intensity of immune activation [32,33]. Canonical models derived largely from *Arabidopsis* describe mutual antagonism between JA- and SA-dependent defense cascades, where SA-rich signaling suppresses JA-responsive outputs and vice versa [34]. Nevertheless, accumulating evidence has documented concurrent elevation of JA- and SA-associated metabolites contributing to enhanced disease resistance in several autoimmune and stress-responsive plant genotypes, illustrating context-dependent synergistic interactions that remain incompletely characterized in soybean and many crop species [35,36]. Here, coordinated accumulation of multiple bioactive JA derivatives and the SA conjugate SAG in *gmdba1* (Figure 7c–e), alongside transcriptomic signatures, indicates that its developmental defects and enhanced disease resistance arise from chronically activated immune-hormone signaling (Figure 1, Figure 5 and Figure 7). It should be noted that our multi-omics results only establish a correlative link between sustained JA/SA activation and the dwarf phenotype of *gmdba1*, rather than definitive causal evidence. Consistent with this, chronic overaccumulation of JA or SA is known to suppress cell proliferation and expansion, inducing stunted growth alongside activated defense responses in diverse autoimmune mutants [8,11,17]. Collectively, these findings indicate that JA/SA signaling concurrently governs both the stunted growth and disease resistance phenotypes of *gmdba1*.

### 3.3. Breeding Implications and Limitations

The major breeding value of the *GmDBA1/2* locus resides in its ability to confer enhanced disease resistance accompanied by readily quantifiable developmental costs, which illustrates a genetic trade-off that breeders need to optimize. The paired-NLR architecture and reproducible phenotype observed in the double-edited line offer a clear target for tuning immune strength, rather than relying on full loss-of-function alleles that trigger severe autoimmune penalties [4,14]. Deployment of genes activating basal immune networks can lessen dependence on discrete pathogen-recognition events and enrich the available resistance genetic resources [2,3,37]. The fine-mapped interval on chromosome 18 and NLR variants uncovered via map-based cloning supply molecular markers to screen natural and induced allelic variation at the *GmDBA1/2* locus. These allelic resources can be exploited to test whether partial-loss-of-function or regulatory haplotypes preserve favorable resistance while circumventing the severe morphological defects observed in the *gmdba1* mutant [2,4]. Given that genetic perturbation at the *GmDBA1/2* locus (as seen in the double-edited material) leads to a pronounced dwarf phenotype, direct application of full-loss-of-function alleles is unlikely to be agronomically desirable. A more practical goal is to uncouple or attenuate resistance and developmental outputs via allele mining, gene editing, or regulatory engineering [14,19].

## 4. Materials and Methods

### 4.1. Plant Materials and Mutagenesis

Hedou 12 (H12) was the wild-type cultivar used for constructing the EMS-mutagenized population. Seeds were treated with 0.6% (*v*/*v*) EMS for 12 h at room temperature, neutralized in 5% sodium thiosulfate solution, thoroughly rinsed under running tap water for around 1 h, air-dried, and subsequently sown. Untreated H12 seeds were planted as wild-type controls. The gmdba1 mutant was identified at the M_4_ generation and underwent repeated self-pollination to perform genetic inheritance assays. Williams 82 (W82) was employed to build the mapping population and acted as the genetic background for CRISPR/Cas9-mediated genome editing.

For field trials under natural sunlight with 70–100 μW cm^−2^ UV-B radiation, H12 and *gmdba1* were cultivated in Qingdao, Shandong (35.35° N, 119.3° E) during the summer growing season. For greenhouse experiments, plants were raised in square pots filled with a 1:1 (*v*/*v*) blend of Pindstrup substrate and vermiculite, with growth parameters set to 25 °C and a 16 h light/8 h dark photoperiod. Developmental phenotypes were scored in either field or greenhouse environments, as indicated in individual figure legends. The *gmdba1* mutant was phenotypically assessed side-by-side with its isogenic wild-type H12 under matched growing conditions. Similarly, CRISPR-edited transgenic lines were assayed alongside the W82 wild-type control. Each mutant or edited genotype and its corresponding wild-type counterpart were grown under strictly identical environmental regimes.

### 4.2. Scanning Electron Microscopy (SEM) Observation

Samples were pre-fixed in pre-cooled glutaraldehyde solution. Vacuum pumping was performed for 10 min using a vacuum pump, and samples were subsequently incubated overnight at 4 °C. Fixed tissues were rinsed five times (10 min per wash) with 1× PBS buffer. Serial ethanol dehydration was carried out in a graded ethanol series (30%, 50%, 60%, 70%, 85%, 95%, and twice with 100% ethanol) for 20 min for each concentration. Dehydrated samples were stored in 100% ethanol at 4 °C, followed by CO_2_ critical-point drying. Dried specimens were preserved in a dry box, mounted on stubs with conductive adhesive tape, and dissected under a stereomicroscope. After gold-sputtering for 2 min, samples were observed and imaged under a scanning electron microscope (Quanta 250 FEG, FEI, Hillsboro, OR, USA).

### 4.3. Phytophthora Sojae Infection Assay

*P. sojae* P6497 was grown on carrot agar at 25 °C in darkness for 7 days. Mycelium-containing agar plugs measuring 3 × 3 mm were excised from the outer growing region of the culture. Detached leaves were placed on agar, and the mycelial surface of a plug was positioned against the leaf before the plate was sealed. For hypocotyl assays, an approximately 1 mm incision was made in dark-grown seedlings, and a mycelium-containing agar plug was applied to the wound. Inoculated material was incubated at 30 °C and relative humidity above 60%. Pathogen biomass in infected soybean tissues was quantified by qRT-PCR. RNA was extracted from infected samples. The relative biomass of *P. sojae* was calculated by comparing the abundance of *PsActin* (pathogen reference) against *GmCYP2* (soybean plant reference). Primer sequences for *PsActin* and *GmCYP2* are listed in Table S3. Three biological replicates were included for each treatment.

### 4.4. Genetic Analysis and Map-Based Cloning

An F_2_ population was generated by crossing W82 and *gmdba1*. Wild-type and dwarf phenotype counts were compared with a 3:1 model using a Pearson chi-square goodness-of-fit test. DNA was extracted from young leaves using a modified CTAB method. Initial genome-wide screening used 21 homozygous F_2_ mutant plants and 52 InDel markers covering the 20 soybean chromosomes (Table S1); regional mapping subsequently used the 94- and 131-plant cohorts described in Section 2.2. Marker genotypes were classified as H12-type (a), W82-type (b), or heterozygous (h). For marker segregation scored among recessive mutant F_2_ plants, recombinant allele frequency was calculated as r = (h + 2b)/[2(a + b + h)] and expressed as a percentage by multiplying by 100. SNP marker PCR products were sequenced for genotyping. Recombinants identified with additional chromosome 18 markers were used to delimit the candidate interval. This linkage analysis maps the phenotype-associated locus rather than determining the separate functions of its linked genes.

### 4.5. Whole Genome Sequencing

Pooled whole-genome resequencing was performed to identify genomic regions associated with the dwarf and wrinkled-leaf phenotype of the *gmdba1* mutant. The *gmdba1* mutant was backcrossed to wild-type soybean cultivar H12 to generate BC_1_F_1_ plants, which were self-pollinated to produce a BC_1_F_2_ segregating population. Phenotypic segregation in the BC_1_F_2_ population followed a 3:1 ratio of wild-type to mutant individuals, consistent with a single recessive genetic locus controlling the mutant phenotype.

Three DNA pools were constructed for whole-genome resequencing: the parental H12 pool (R01) and the BC_1_F_2_ mutant phenotypic pool (R02). Each pool contained equally mixed genomic DNA from 30 individual plants with uniform growth status. Sequencing libraries were constructed and sequenced on the Illumina NovaSeq platform using paired-end 150 bp (PE150) reads, with an average sequencing depth of 30× for each pool.

Clean reads were aligned to the soybean reference genome Williams 82 (Wm82.a2.v1, Gmax_275) using BWA (v0.7.17) software. SNP calling and stringent filtering were performed using SAMtools (v1.12) and GATK (v4.2.0) pipelines. The SNP-index was calculated at each genomic position for all pools, and the ΔSNP-index was determined by subtracting the SNP-index of the wild-type pool from that of the mutant pool. Genomic intervals with a sustained ΔSNP-index ≥ 0.7 were defined as candidate regions tightly linked to the causal locus of *gmdba1*. These pooled whole-genome resequencing results provided reliable candidate intervals for subsequent fine-mapping and gene cloning. This experiment was conducted by Berry Genomics (Beijing, China).

### 4.6. CRISPR/Cas9 Vector Construction and Transformation

A guide construct was designed to target *Glyma.18g226500* and *Glyma.18g226800* simultaneously. The reported target sequence, including its adjacent sgRNA, is GATGCTGCTTTGTTAAACACTGG, and TGG is the PAM site. The construct was introduced into W82 by *Agrobacterium tumefaciens* EHA105-mediated cotyledonary-node transformation performed by Weimi Biotechnology (Changsha, China). T_0_ transformants were genotyped by sequencing. One homozygous T_2_ line carrying deletions in both candidate genes was selected for developmental and P6497 inoculation assays. This material is described as a double-edited line because complete loss of native protein function was not directly established.

### 4.7. Transcriptome Sequencing Analysis (RNA-Seq)

For transcriptome RNA-seq analysis, apical shoot tissues were harvested from wild-type H12 and the dwarf *gmdba1* mutant at the V_6_ vegetative growth stage. Plants exhibiting uniform growth performance were chosen for sampling, with three independent biological replicates per genotype to guarantee biological reproducibility. Total RNA was isolated and subjected to strict RNA quality assessment; RNA samples meeting quality thresholds were submitted to BGI Genomics for paired-end high-throughput transcriptome sequencing with an average sequencing depth of 6 Gb per sample.

Raw sequencing reads were pre-processed using SOAPnuke (v1.5.2) to filter low-quality data: adapter-contaminated reads, reads containing more than 10% ambiguous N bases, and reads where over 50% of bases possessed a Phred quality score ≤ 15 were discarded. The resulting high-quality clean reads were saved in FASTQ format. Clean reads were aligned against the soybean Williams 82 reference genome assembly Wm82.a2.v1 using HISAT2 (v2.1.0) to obtain genome-mapping statistics, including the percentage of uniquely mapped reads. For gene-level mapping, clean reads were mapped to reference gene sequences with Bowtie2 (v2.2.5). Gene-level expression abundances were quantified and normalized to TPM values by RSEM (v1.2.8).

Gene functional annotation of assembled unigenes was performed against seven public databases: GO, KEGG, NR, NT, Swiss-Prot, Pfam, and KOG; transcription-factor prediction was also conducted. Within-group differential expression analysis was implemented using DESeq, which employs a negative-binomial generalized linear model. Multiple-testing correction was performed to generate adjusted *p*-values. Genes satisfying thresholds of fold change ≥ 2.00 and adjusted *p*-value ≤ 0.001 were regarded as differentially expressed genes (DEGs). Between-group DEG detection was carried out via PossionDis with criteria fold change ≥ 2.00 and FDR ≤ 0.001. The R-based pheatmap function was applied to generate expression-clustering heatmaps of DEG sets. Functional enrichment for DEGs was completed using phyper for KEGG enrichment and the TermFinder R package (v0.1.0) for GO enrichment analysis; terms with Q-value ≤ 0.05 were considered significantly enriched.

Time-series expression clustering was performed by Mfuzz (v2.34.0), and weighted gene co-expression network analysis was conducted using the WGCNA (v1.48) R package to further dissect transcriptome reprogramming between wild-type H12 and the *gmdba1* mutant.

### 4.8. RNA Extraction and RT-qPCR

Total RNA was extracted from soybean tissues using TRIzol reagent (Mei5bio, Beijing, China, MF034). First-strand cDNA was synthesized from total RNA with HiScript III RT SuperMix (Vazyme, Nanjing, China, R433-01) following the manufacturer’s instructions.

RT-qPCR reactions were performed on an Applied Biosystems (Foster City, CA, USA) 7500 Real-Time PCR System using SYBR Green-based real-time fluorescence quantitative PCR. Soybean ACTIN was selected as the internal reference gene. Gene-specific primers were designed to span introns to avoid amplification of genomic DNA.

The relative gene expression levels were calculated using the 2^^(−ΔΔCt)^ method [38]. Briefly, ΔC_t_ = Ct(target) − C_t_(ACTIN); ΔΔC_t_ = ΔCt(sample) − ΔC_t_(calibrator). Three independent biological replicates with three technical replicates each were included for all assays. All primer sequences used for qRT-PCR are in Table S3.

### 4.9. LC-MS/MS Hormone Detection and Analysis

Plant samples were ground into powder with a grinder in liquid nitrogen. Approximately 50 mg fresh weight of tissue powder was mixed with internal standard solution and methanol–water–formic acid extraction buffer. After vortexing and centrifugation at 12,000 r/min for 5 min at 4 °C, the supernatant was concentrated and redissolved in 80% methanol solution, followed by filtration through a 0.22 μm membrane for LC-MS/MS detection. Metabolite profiling was performed using the ExionLC™ AD UPLC system coupled with a QTRAP^®^ 6500+ tandem mass spectrometer (AB Sciex, Concord, ON, Canada). Gradient elution was conducted with acidified ultrapure water and acetonitrile. The ESI source was operated in positive and negative ionization modes with optimized instrumental parameters. Based on the self-built MWDB database and MRM scanning mode, metabolite identification and accurate quantitative analysis were completed by chromatographic peak integration and standard curve calibration, and all assays were commissioned by Metware Biotechnology (Wuhan, China).

### 4.10. Phylogenetic Analysis

Homologous protein sequences of GmDBA1/2 across representative plant taxa were downloaded from the Phytozome online resource. Sequence multiple alignment was implemented via MUSCLE under default settings. A neighbor-joining phylogenetic tree was built within MEGA11 software (v11.0.13); the Poisson correction model was selected, and the pairwise-deletion mode was adopted to handle gap positions and missing amino-acid information. Bootstrap resampling with 1000 replicates was carried out to assess the confidence of each branching node, and corresponding bootstrap supports were annotated onto tree branches. Branch lengths correspond to evolutionary distances, measured as the number of amino-acid substitutions per residue site.

### 4.11. Statistical Analysis

All experiments were carried out with three biological replicates and three technical replicates. Student’s *t*-test was used for comparisons between two groups, while one-way ANOVA followed by Tukey’s or LSD post hoc test was adopted for multiple-group comparisons. Statistical analyses were performed in GraphPad Prism (v8.0.2) or Minitab (v18.1).

## 5. Conclusions

Genetic mapping and sequence analysis prioritized *GmDBA1* and *GmDBA2* within a chromosome-18 locus linked to the recessive *gmdba1* phenotype. Both the original EMS-derived mutant and one CRISPR-mediated double-edited line in the W82 background exhibited dwarfism as well as attenuated disease symptoms upon inoculation with *P. sojae* isolate P6497. Transcriptomic profiling revealed widespread defense-related transcriptional reprogramming, which was accompanied by elevated accumulation of JA- and SA-derived metabolites in the mutant. Collectively, our study identifies these two NLR genes as promising candidates for dissecting the molecular interplay between plant growth and immunity. The mutant genetic materials reported here furnish valuable resources for further mechanistic investigation of soybean immune responses, and *GmDBA1*/*GmDBA2* represent potential target genes for future molecular breeding aiming to improve *Phytophthora* resistance in soybean.

## Data Availability

The original data presented in the study are openly available in GSA project accessions at RNA-seq: PRJCA076483 and WGS-seq: PRJCA076439.

## Supplementary Materials

The following supporting information can be downloaded at: https://www.mdpi.com/article/10.3390/plants15213296/s1, Table S1, molecular markers and primer; Table S2, the 22 annotated genes in the mapped interval; Table S3, Primers used for qPCR; Table S4, hormone content; Table S5, *GmDBA1* and *GmDBA2* SNP.

## Appendix A

Additional mapping, sequence-comparison, and transcriptomic figures are provided below.
